# SPOCK2 modulates neuropathic pain by interacting with MT1-MMP to regulate astrocytic MMP-2 activation in rats with chronic constriction injury

**DOI:** 10.1186/s12974-024-03051-5

**Published:** 2024-02-22

**Authors:** Chenglong Wang, Yitong Xu, Miao Xu, Cong Sun, Xiaojiao Zhang, Xueshu Tao, Tao Song

**Affiliations:** 1 Department of Pain, The First Hospital of China Medical University, Shenyang, 110001 China; 2 Department of Pathology, The First Hospital of China Medical University, Shenyang, 110001 China

**Keywords:** SPOCK2, Neuropathic pain, Astrocyte, CCI, MMP-2, MT1-MMP

## Abstract

**Background:**

Neuropathic pain (NP) is a kind of intractable pain. The pathogenesis of NP remains a complicated issue for pain management practitioners. SPARC/osteonectin, CWCV, and Kazal-like domains proteoglycan 2 (SPOCK2) are members of the SPOCK family that play a significant role in the development of the central nervous system. In this study, we investigated the role of SPOCK2 in the development of NP in a rat model of chronic constriction injury (CCI).

**Methods:**

Sprague–Dawley rats were randomly grouped to establish CCI models. We examined the effects of SPOCK2 on pain hpersensitivity and spinal astrocyte activation after CCI-induced NP. Paw withdrawal threshold (PWT) and paw withdrawal latency (PWL) were used to reflects the pain behavioral degree. Molecular mechanisms involved in SPOCK2-mediated NP in vivo were examined by western blot analysis, immunofluorescence, immunohistochemistry, and co-immunoprecipitation. In addition, we examined the SPOCK2-mediated potential protein–protein interaction (PPI) in vitro coimmunoprecipitation (Co-IP) experiments*.*

**Results:**

We founded the expression level of SPOCK2 in rat spinal cord was markedly increased after CCI-induced NP, while SPOCK2 downregulation could partially relieve pain caused by CCI. Our research showed that SPOCK2 expressed significantly increase in spinal astrocytes when CCI-induced NP. In addition, SPOCK2 could act as an upstream signaling molecule to regulate the activation of matrix metalloproteinase-2 (MMP-2), thus affecting astrocytic ERK1/2 activation and interleukin (IL)-1β production in the development of NP. Moreover, in vitro coimmunoprecipitation (Co-IP) experiments showed that SPOCK2 could interact with membrane-type 1 matrix metalloproteinase (MT1-MMP/MMP14) to regulate MMP-2 activation by the SPARC extracellular (SPARC_EC) domain.

**Conclusions:**

Research shows that SPOCK2 can interact with MT1-MMP to regulate MMP-2 activation, thus affecting astrocytic ERK1/2 activation and IL-1β production to achieve positive promotion of NP.

## Background

Neuropathic pain (NP) is an intractable pain caused by a lesion or disease affecting the somatosensory system [1]. Traditional analgesics have poor efficacy and are prone to adverse reactions, such as overdose toxicity and opioid dependence. Therefore, the pathogenesis of NP remains a complicated issue for pain management practitioners.

A large number of literatures have confirmed that the activation of microglia and astrocytes in the spinal cord is closely related to the generation of NP [2–10]. Nerve injury can lead to the activation of microglia and astrocytes and cause the release of inflammatory mediators (pro-inflammatory factors, chemokines, cytokines, etc.), leading to the production of NP by peripheral and central nervous system sensitization [11, 12]. In the early stages after nerve injury, microglial activation is important for the induction of NP [13]. However, in late stages, the activation of astrocytes in the spinal cord is critical for NP maintenance [14, 15].

SPARC/osteonectin, CWCV, and Kazal-like domains proteoglycan 2 (SPOCK2) is a secreted multidomain proteoglycan belonging to the BM-40/SPARC family of extracellular proteins [16]. It is expressed in brain endothelial cells and neurons, and it is part of the extracellular matrix [17]. The SPOCK2 gene encodes a full-length 424-amino acid protein containing a thyroglobulin-1, a follicle-like domain, and a calcium-binding domain. Currently, few functional studies have focused on SPOCK2, which showed that SPOCK2 was associated with lung adenocarcinoma [18], prostate cancer [19], ovarian cancer [20], and bronchopulmonary dysplasia [21]. In a recent genome-wide association study (GWAS) [22] of back pain (BP) combining data from the UK Biobank and CHARGE Consortium databases, three BP-associated loci, including one novel SPOCK2 gene region, were identified and replicated. In addition, another GWAS analysis of BP [23] showed that SPOCK2 gene can contribute to chronic but not acute BP. BP is usually caused by spinal nerve inflammation, and chronic BP can produce central sensitization and evolve into NP. Therefore, the present study aimed to investigate the role of SPOCK2 in the pathogenesis of NP using a well-characterized rat model of chronic constriction injury (CCI).

Despite the limited research on SPOCK2 function, current evidence indicates that SPOCK family proteins are involved in the regulation of matrix metalloproteinases (MMPs). For example, Ren et al. [24] found that SPOCK2 affects endometrial cancer cell behavior through the regulation of membrane-type 1 matrix metalloproteinase (MT1-MMP/MMP-14) and matrix metalloproteinase 2 (MMP-2). Also, Nakada et al. [25, 26] showed that both SPOCK1 and SPOCK3 could inhibit MT1-MMP-mediated activation of MMP-2, but SPOCK2 abolished MT1-MMP inhibition. MMPs are extensively involved in neuroinflammation and tissue remodeling through the cleavage of extracellular matrix proteins, cytokines, and chemokines [27–29]. Both MT1-MMP and MMP-2 are important members of the MMPs family, and they are closely involved in NP development [27, 30, 31]. Moreover, in a glioma-related study [32], we founded that SPOCK2 could be associated with astrocytic activation. According to the above research, we hypothesized that SPOCK2 may be related to MMP-2 and astrocytic activation in the development of NP. This study aimed to identify the role of SPOCK2 in the development of NP and its underlying mechanism.

## Methods

### Animals

Adult male Sprague–Dawley rats weighing 250–300 g were purchased from SPF Biotechnology Co., Ltd. (Beijing, China). The rats were housed under a 12/12 h light/dark cycle at 23 ± 1 ℃ with free access to food and water. The Animal Care and Use Committee of China Medical University approved all procedures used in the study (IACUC no. CMU2022004).

### NP model induction

The CCI rat model of NP was established according to procedures described by Bennett and Xie [33]. Firstly, rats were anesthetized with 1% isoflurane, and a 1.5 cm lateral incision was made in the right hindlimb. The muscle was bluntly separated to expose the sciatic nerve trunk. Secondly, the sciatic nerve trunk was loosely ligated by four silks (4–0#) at an interval distance of 1 mm. The sutures were gently tightened until a brisk twitch in the right hindlimb was observed. Finally, layered skin suturing was performed.

### Mechanical allodynia (von Frey test)

Mechanical allodynia was assessed by measuring paw withdrawal threshold (PWT) in response to von Frey filament stimulation. The rats were placed in a transparent plastic cage with a mesh bottom for 20 min before testing. PWT was assessed using a dynamic plantar esthesiometer (Ugo Basile, 37,450, Italy). A probe was applied to the mid-plantar surface of the hind paw under increasing pressure. The cut-off pressure was set to 50 g, and the esthesiometer automatically recorded the force causing the withdrawal response. For each animal, ≥ 3 measurements were performed every 10-min to stimulate each hind paw.

### Thermal hyperalgesia (hot plate test)

Thermal hyperalgesia was assessed by measuring paw withdrawal latency (PWL) in response to hot plate test. A hot plate analgesia meter (Ugo Basile, 35,300, Italy) was prepared with a pre-set plate temperature of 53 ± 1 °C. Once the rat was placed on the hot plate, the time between placement and licking, shaking, or stepping of the hindpaws was recorded. The cut-off time was set to 30 s to avoid tissue damage. For each animal, ≥ 3 measurements were performed every 10 min.

### Drugs and administration

The effective and selective MMP-2 inhibitor, ARP 100 (MMP-2 inhibitor III, compound 10a), was purchased from Selleck Chemicals (Shanghai, China). The MMP-2 inhibitor was dissolved in dimethyl sulfoxide (DMSO) to a concentration of 1 nmol/µL. The doses of MMP-2 inhibitor were chosen as previously described [30]. The in vivo SPOCK2-siRNA (2 Ome + 5 Chol) was commercially synthesized by RiboBio Co. Ltd. (Guangzhou, China). The specific target sequence of SPOCK2-siRNA was 5′-GTGAGAACTCGAAGCAGAA-3′. Negative control siRNA (NC-siRNA) was synthesized using a scrambled sequence of nucleotides. After methylation and cholesterol modification, in vivo siRNA can be stably transfected in vivo and have high transfection efficiency without transfection reagents [34].The freeze-dried siRNA powder was dissolved in 0.9% normal saline (NS). The method of administration was chosen as previously described [35]. The recombinant lentiviral vectors carrying SPOCK-shRNA (pGLV-3-GFP-SPOCK2-shRNA) was purchased from GenePharma Co. Ltd. (Shanghai, China). The target sequence of SPOCK2-shRNA was 5′-GUGAGAACUCGAAGCAGAA-3′, and the negative control shRNA vector with a scrambled shRNA (shScr) insert was used to control any effects caused by non-RNAi mechanisms. The recombinant SPOCK2 protein (rSPOCK2) was purchased from R&D Systems (Minneapolis, MN). The rSPOCK2 was dissolved in 0.9% NS to a concentration of 0.1 ng/µL. The doses and administration of rSPOCK2 were based on previous literature [8]. Drugs or vehicles (DMSO, NS) were delivered into the cerebrospinal fluid space around the lumbosacral spinal cord through intrathecal (i.t.) administration.

### Experimental protocols

#### Protocol I

**Fig. 1:**
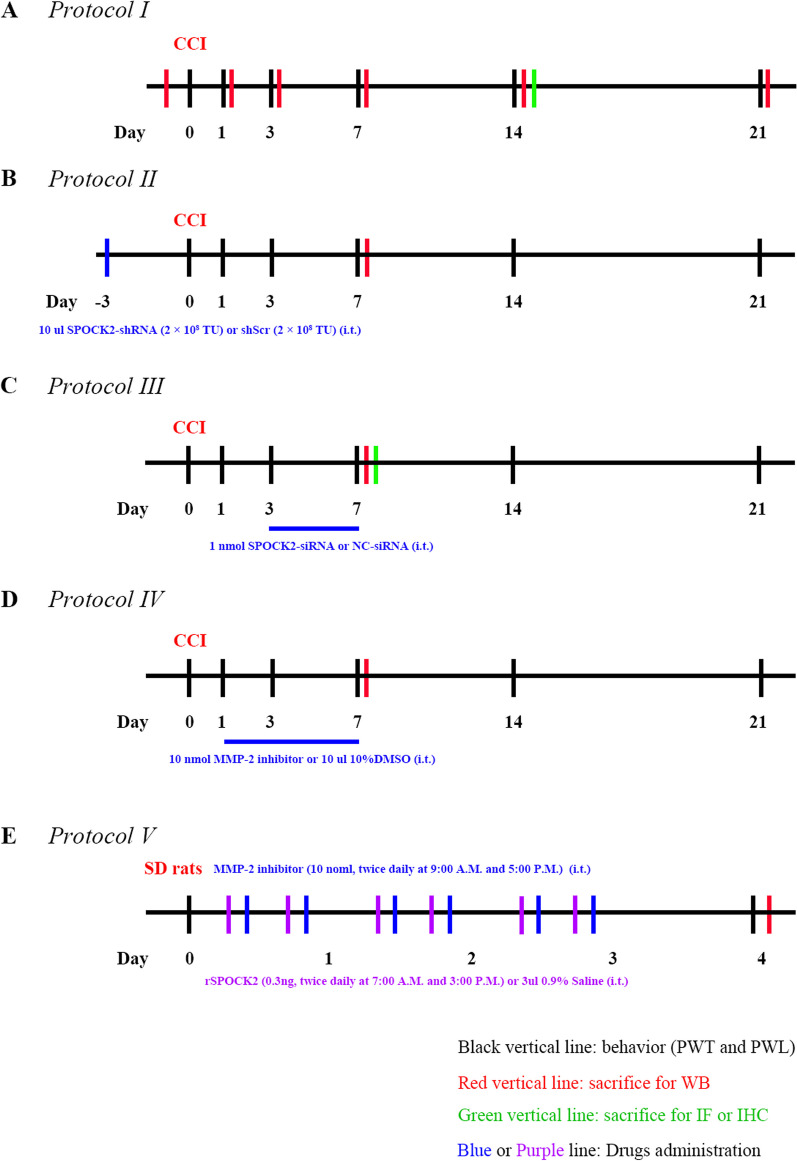
Schematic illustration of the experimental protocols

To test the time course of changes in pain behaviors and expression of SPOCK2 in the spinal cord, rats were assigned to sham (n = 5) and CCI (n = 30) groups. The CCI group underwent CCI surgery, while the same procedure was performed in the sham group, except for the ligation of the sciatic nerve. PWT and PWL were assessed at the ipsilateral hind paws 24 h prior to CCI and day 1, 3, 7, 14 and 21 after CCI. Sham rats were sacrificed at final time point and five CCI rats were sacrificed after PWT and PWL measurement at each time point (Fig. 1A). The protein was extracted from L4-6 rat spinal cord of ipsilateral side for western blot analysis. Additional CCI rats used for immunofluorescence and immunohistochemistry were sacrificed at 14 days after CCI.

#### Protocol II

To explore the role of SPOCK2 in the spinal cord in the process of pain occurrence, rats were divided into 4 groups (n = 5 for each group): sham; CCI; CCI treated with shScr (CCI + shScr); CCI treated with SPOCK2-shRNA (CCI + SPOCK2-shRNA). 10ul SPOCK2-shRNA (2 × 10^8^ TU) or 10ul scrambled shRNA (2 × 10^8^ TU) was i.t. injected 3 days prior to the beginning of CCI. PWT and PWL were assessed at the ipsilateral hind paws 24 h prior to CCI and day 1, 3, 7, 14 and 21 after CCI, and rats were sacrificed at the end of the protocol. Another four groups (n = 5 for each group) rats used for western blot analysis were sacrificed at 7 days after CCI (Fig. 1B).

#### Protocol III

To further examine the role and mechanism of SPOCK2 in the development of NP, rats were divided into 4 groups (n = 5 for each group): sham; CCI; CCI treated with NC-siRNA (CCI + NC-siRNA); CCI treated with SPOCK2-siRNA (CCI + SPOCK2-siRNA). 1 nmol SPOCK2-siRNA or NC-siRNA was i.t. injected 5 continuous days (after CCI, 3–7 days). PWT and PWL were assessed at the ipsilateral hind paws 24 h prior to CCI and day 1, 3, 7, 14 and 21 after CCI, and rats were sacrificed at the end of the protocol. Another four groups (n = 5 for each group) rats used for western blot analysis were sacrificed at 7 days after CCI. Additional CCI rats treated with NC-siRNA and SPOCK2-siRNA used for immunofluorescence were sacrificed at 7 days after CCI (Fig. 1C).

#### Protocol IV

To further study the relationship between SPOCK2 and MMP-2 in the development of NP, rats were divided into 4 groups (n = 5 for each group): sham; CCI; CCI treated with 10%DMSO (vehicle) (CCI + 10%DMSO); CCI treated with MMP-2 inhibitor (CCI + MMP-2 inhibitor). 10ul 10%DMSO or 10 nmol MMP-2 inhibitor was i.t. injected 7 continuous days (after CCI, 1–7 days). PWT and PWL were assessed at the ipsilateral hind paws 24 h prior to CCI and day 1, 3, 7, 14 and 21 after CCI, and rats were sacrificed at the end of the protocol. Another four groups (n = 5 for each group) rats used for western blot analysis were sacrificed at 7 days after CCI (Fig. 1D).

#### Protocol V

To further study the role of SPOCK2 involved in NP and the relationship between SPOCK2 and MMP-2 in the development of NP, rats were divided into 3 groups (n = 5 for each group): SD rats treated with 0.9% NS (Vehicle); SD rats treated with rSPOCK2 (rSPOCK2); SD rats treated with rSPOCK2 and MMP-2 inhibitors (rSPOCK2 + MMP-2 inhibitor). We i.t. injected rSPOCK2 (0.3 ng, twice daily at 7:00 A.M. and 3:00 P.M.) or 3ul 0.9%NS (Vehicle) for 3 days. In the rSPOCK2 + MMP-2 inhibitor group, MMP-2 inhibitor was i.t. injected (10 nmol, twice daily at 9:00 A.M. and 5:00 P.M.) for 3 days. 16 h (day 4) after the end of drug administration, PWT and PWL were evaluated. Rats used for western blot analysis were sacrificed at the end of the protocol (Fig. 1E).

### Western blot

Isolated rat lumbar spinal cord (L4–6) was homogenized in an ice-cold lysis buffer containing a protease and phosphatase inhibitor cocktail. Supernatants were collected by centrifugation at 12,000 ×*g* for 15 min at 4 °C. Total protein was obtained from the spinal cord using protein extraction kits, separated by 8% sodium dodecyl sulfate–polyacrylamide gel electrophoresis, and transferred onto polyvinylidene difluoride (PVDF) membranes. The membranes were next placed in blocking buffer (5% milk in Tris-buffered saline with Tween-20) for 1 h and incubated overnight (16–18 h) at 4 °C with primary antibodies against SPOCK2 (1:1,000, Cat# 217,044, RRID: AB_3076329, Abcam, Cambridge, UK), MT1-MMP (1:1,000, Cat# 14,552–1-AP, RRID: AB_2250751, Proteintech, Wuhan, China), MMP-2 (1:1,000, Cat# 66,366–1-Ig, RRID: AB_2881746, Proteintech), ERK1/2 (1:2,000, Cat# 9102, RRID: AB_330744, Cell Signaling Technology, Danvers, MA, USA), p-ERK1/2 (1:1,000, Cat# 4370, RRID: AB_2315112, Cell Signaling Technology), interleukin-1β (IL-1β) (1:1,000, Cat# sc-7884, RRID: AB_2124476, Santa Cruz, TX, USA), CC-chemokine ligand 2 (CCL2) (1:2000, Cat# ab7202, RRID: AB_305755, Abcam), JNK (1:3,000, Cat# 17,572–1-AP, RRID: AB_2266214, Proteintech), p-JNK (1:2,000, Cat# 80,024–1-RR, RRID: AB_2882943, Proteintech), and glyceraldehyde-3-phosphate dehydrogenase (GAPDH) (1:10,000, Cat# 60,004–1-Ig, RRID: AB_2107436, Proteintech). After washing three times with TBST for 5 min, the membranes were incubated with horseradish peroxidase (HRP)-conjugated secondary antibodies (anti-rabbit, 1:10,000, Cat# SA00001-2, RRID: AB_2722564, Proteintech) (anti-mouse, 1:10,000, Cat# SA00001-1, RRID: AB_2722565, Proteintech) for 1 h at room temperature. The labeled proteins were visualized and quantified using an enhanced chemiluminescence detection system.

### Immunofluorescence

Frozen immunofluorescence sections were used for these experiments. Briefly, rats were transcardially perfused with 0.9% normal saline (NS), followed by 4% paraformaldehyde. Spinal cords were dissected, fixed overnight in 4% paraformaldehyde at 4 °C, and then continuously dehydrated in 20% and 30% sucrose for 24 h each. Spinal cord sections were sliced 10-µm thick with a cryostat. Following non-specific binding with 5% goat serum in 0.3% Triton for 1 h at room temperature, the sections were incubated overnight (16–18 h) at 4 °C with primary antibodies against SPOCK2 (1:200, Cat# 217,044, RRID: AB_3076329, Abcam), MT1-MMP (1:200, Cat# 14,552–1-AP, RRID: AB_2250751, Proteintech), MMP-2 (1:200, Cat# 66,366–1-Ig, RRID: AB_2881746, Proteintech), glial fibrillary acidic protein (GFAP) (1:400, Cat# 3670, RRID: AB_561049, Cell Signaling Technology), and monoclonal mouse anti-RAT CD11b (1:400, Cat# MCA275R, RRID: AB_321302, BIO-RAD, USA). On the second day, the sections were incubated for 1 h at room temperature with the following secondary antibodies: Alexa Fluor 488 goat anti-rabbit (1:400, Cat# A-11008, RRID: AB_143165, Thermo Fisher Scientific, USA) and Alexa Fluor 568 goat anti-mouse (1:400, Cat# A-11031, RRID: AB_144696, Thermo Fisher Scientific). A confocal microscope (FV3000, Olympus, Japan) was used to capture all images. Quantitative immunofluorescence analysis was calculated by Image J Software (National Institutes of Health, Bethesda, USA). The positive cells were counted in a 500 um × 500 um measuring frame. Cell counts were then used to determine the total number of positive cells per square millimeter.

### Immunohistochemistry

Immunohistochemical paraffin sections were used. Briefly, rat spinal cords were isolated following transcardial perfusion in 0.9% NS and 4% paraformaldehyde, post-fixed, and embedded in paraffin. Spinal cord 4 µm-sections were obtained and next were deparaffinized in xylene and rehydrated in graded ethanol. After the non-specific binding was blocked with 5% goat serum in 0.3% Triton for 1 h at room temperature, the sections were incubated overnight (16–18 h) at 4 °C with primary antibodies against SPOCK2 (1:200, Cat# 217,044, RRID: AB_3076329, Abcam). The signal was visualized using Elivision Super HRP IHC Kits (Maixin-Bio, China) and 3,3-diaminobenzidine (Maixin-Bio, China), and the nuclei were counterstained with hematoxylin.

### Cell culture and plasmids transfection

HEK-293 T cells were purchased from the Shanghai Cell Bank (GNHu17, Shanghai, China) and cultured in high-glucose Dulbecco’s modified Eagle’s medium (Invitrogen, 11,960,044) with 10% fetal bovine serum (FBS; FB15015; Clark Biosciences, USA), 1% Glutamax (Invitrogen, 35,050,061), and 1% Sodium Pyruvate 100 mM Solution (Invitrogen, 11,360,070) at 37℃ and 5% CO_2_.

The wild-type (WT) plasmid (pCMV6-SPOCK2-Myc-DDK) and control (empty) plasmid (pCMV6-Myc-DDK) were purchased from OriGene (Rockville, MD, USA). ΔKAZAL (mut1) plasmid (pCMV6-ΔKAZAL-myc-DDK), ΔSPARC_EC (mut2) plasmid (pCMV6-ΔSPARC_EC-myc-DDK), and ΔΔ (mut3) plasmid (pCMV6-ΔΔ-myc-DDK), containing both knockout parts of the ΔKAZAL and ΔSPARC_EC plasmids, were obtained from TSINGKE Biological Technology (Beijing, China). Lipofectamine 3000 (Invitrogen, Waltham, MA, USA) was used for plasmid transfection according to the manufacturer’s instructions.

### Coimmunoprecipitation (Co-IP)

HEK-293 T cells were plated in 10 cm dishes. When 90% confluency was reached, the cells were lysed for the assays. Co-IP assays were performed with an Immunoprecipitation Kit consisting of Protein A + G Magnetic Beads (P2179S, Beyotime Biosciences) according to manufacturer’s instructions.

### Data analysis

Data analyses were performed using the GraphPrism 9.0 software (Graph Pad Software, San Diego, CA, USA). All data are presented as mean ± SEM. Image J Software (National Institutes of Health, Bethesda, USA) was used to process the density of the western blot bands and quantitative immunofluorescence analysis. The normal distribution of data was analyzed using the D’Agostino and Pearson test (p > 0.05). Correlation was analyzed using the Pearson correlation test. Differences between groups were compared by a one-way or two-way analysis of variance (ANOVA) followed by Bonferroni’s post hoc test. Statistical significance was set at *p < 0.05 or **p < 0.01.

## Results

### SPOCK2 increase in rats spinal cord after CCI-induced NP

**Fig. 2:**
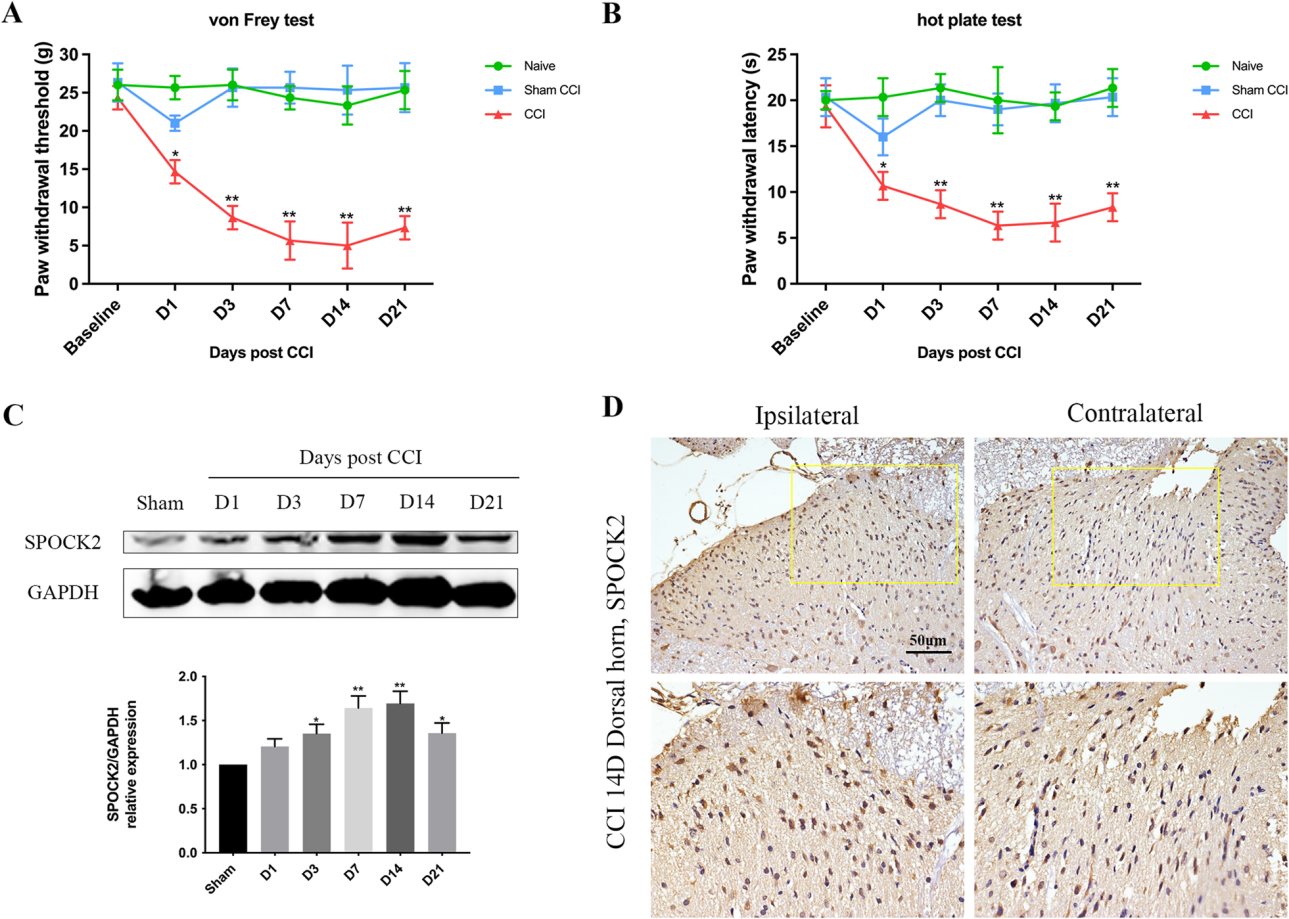
Changes in SPOCK2 expression and pain behaviors in rats after CCI. **A**, **B** PWT and PWL at baseline (day 0) and 1, 3, 7, 14, and 21 days after CCI or sham operation (*P < 0.05, **P < 0.01 vs baseline). **C** Time course of changes in SPOCK2 expression in the spinal cord in the sham group at 1, 3, 7, 14, and 21 days after CCI operation (*P < 0.05, **P < 0.01 vs sham). **D** Immunohistochemistry showing SPOCK2 localization in the spinal dorsal horn and increased ipsilateral expression at 14 days after CCI operation (Scale 50 um). Below column of images: higher power magnification of a section of the image on the top

To explore the role of SPOCK2 in NP, chronic constriction injury (CCI) sugery in rat were used to create NP models as the basis for subsequent research. As described in *Protocol I*, after CCI-induced NP, the mechanical allodynia (PWT) and thermal hyperalgesia (PWL) decreased gradually, indicating successful modeling. As shown in Fig. 2A and B, PWT and PWL decreasing significantly at 7 and 14 days after CCI (p < 0.01). SPOCK2 expression in the rat spinal cord was detected after CCI using western blot analysis and immunohistochemistry. As shown in Fig. 2C, increasing SPOCK2 protein levels are expressed in the rat spinal cord at 7 and 14 days after CCI-induced NP (p < 0.05). At 14 days after CCI, immunohistochemistry of spinal sections showed that SPOCK2 expression on the ipsilateral dorsal horn was markedly stronger than that on the contralateral side (Fig. 2D). The expression level of SPOCK2 protein reached its peak on 7 and 14 days after CCI surgery, which coincides with the occurrence of extreme pain changes increasing, this result leads us to speculate that SPOCK2 plays a certain role in NP rather than coincidentally.

### SPOCK2 is required for NP

**Fig. 3:**
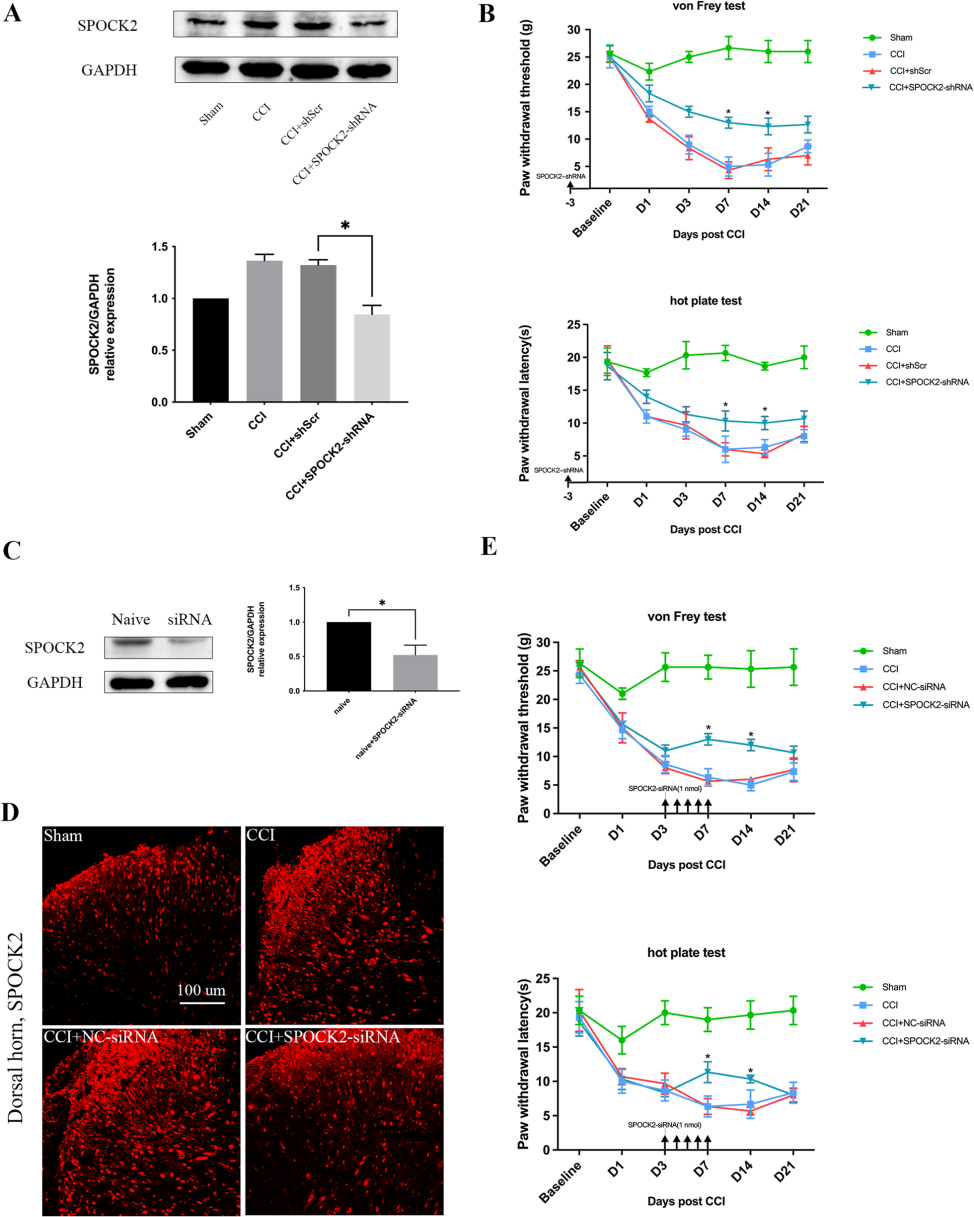
SPOCK2 downregulation alleviates CCI-induced NP. **A** Decreased expression level of SPOCK2 after i.t. injection of SPOCK2-shRNA in rats spinal cord after CCI-induced NP. (*P < 0.05 vs CCI + shScr). **B** i.t. injection of SPOCK2-shRNA 3 days prior to the beginning of CCI increased PWT and PWL at 7 and 14 days after CCI (*P < 0.05 vs CCI + shScr). **C** Western blot analysis showing the knockdown efficacy of SPOCK2-siRNA (*P < 0.05 vs naïve). **D** Immunofluorescence showing decreased SPOCK2 expression in the dorsal horn after i.t. injection of SPOCK2-siRNA (Scale 50 um). **E** Continuous i.t. injection of SPOCK2-siRNA for 5 days (from CCI 3 days to 7 days) increased PWT and PWL at 7 and 14 days after CCI (*P < 0.05 vs CCI + NC-siRNA)

Whether SPOCK2 in the spinal cord could participate in pain hypersensitivity was the first step for this whole building. Knocking down the expression of SPOCK2 using lentiviral vectors carrying SPOCK2-shRNA were used to detect the effect of SPOCK2 on the induction of NP behaviors. We intrathecally (i.t.) injected SPOCK2-shRNA to downregulate SPOCK2 expression in the rat spinal cord as described in *Protocol II*. As shown in Fig. 3A, SPOCK2 expression in the rat spinal cord significantly decreased (p < 0.05) in the CCI + SPOCK2-shRNA group. In addition, PWT and PWL on check points showed the degree of increase (p < 0.05) in mechanical allodynia and thermal hyperalgesia (Fig. 3B). These results indicate that SPOCK2 in the spinal cord can enhance CCI-induced pain hypersensitivity.

After clarifying the involvement of SPOCK2 in the process of pain occurrence, we speculate whether the reduction of SPOCK2 can also reverse pain after it occurs. We intrathecally (i.t.) injected SPOCK2-siRNA to downregulate SPOCK2 expression in the rat spinal cord as described in *Protocol III*. First, we tested the efficiency of SPOCK2-siRNA knockdown in normal Sprague Dawley rats. As shown in Fig. 3C, i.t. injection of SPOCK2-siRNA markedly decreased SPOCK2 expression in the rat spinal cord (p < 0.05), as measured by western blot analysis. After i.t. injection of 1 nmol SPOCK2-siRNA for five continuous days (after CCI, 3–7 days), SPOCK2 expression in the rat spinal cord significantly decreased in the CCI + SPOCK2-siRNA group (Fig. 3D) accompanied by a significant increase (p < 0.05) in mechanical allodynia (PWT) and thermal hyperalgesia (PWL) (Fig. 3E). However, i.t. injection of NC-siRNA showed no changes on the behavior tests.

### SPOCK2 modulates NP by affecting the activation of astrocyte

**Fig. 4:**
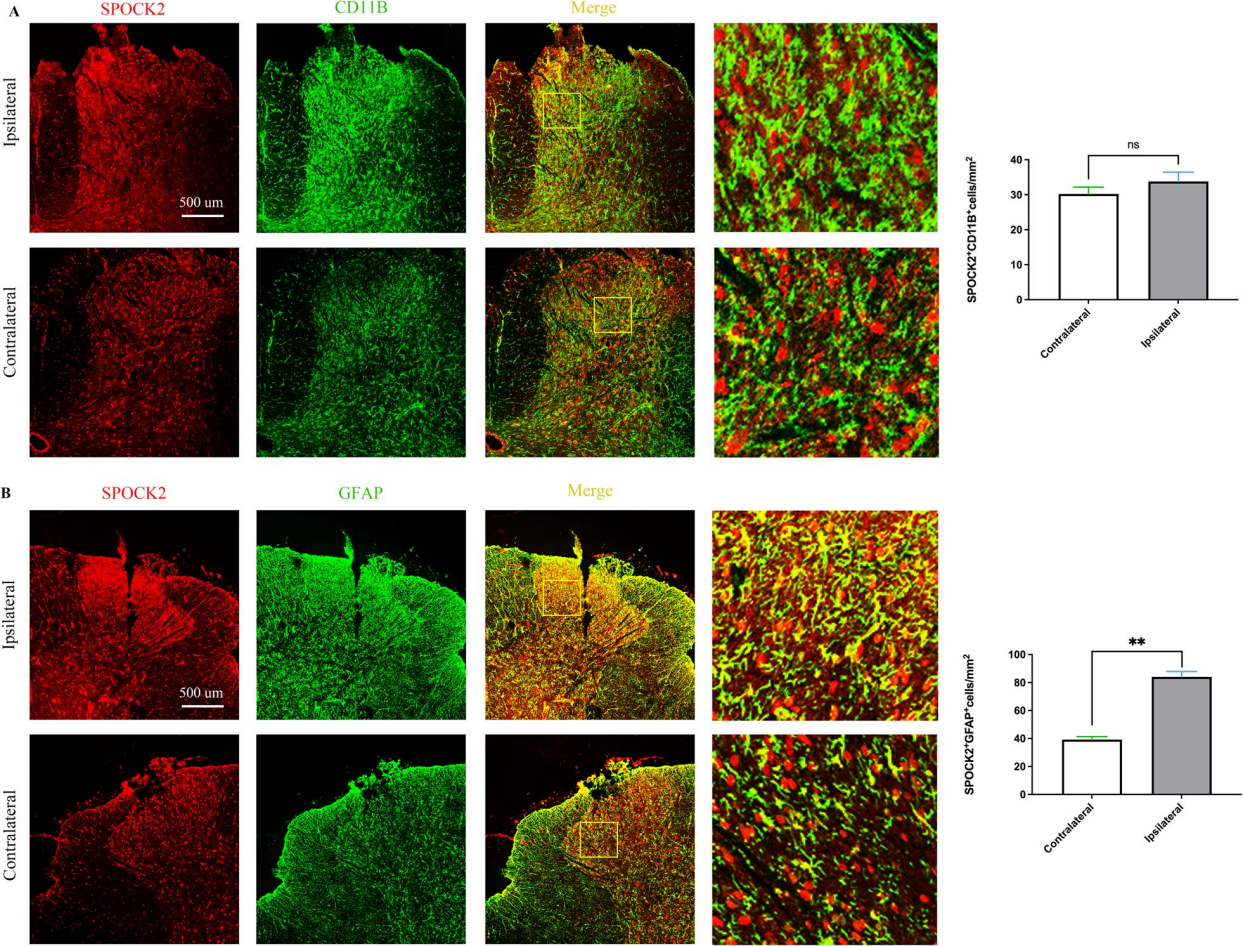
Changes of subcellular (microglia and astrocyte) colocalization of SPOCK2 in rats after CCI. **A** Double staining showed that the changes of SPOCK2 expression in microglia (CD11B) was not significant on the ipsilateral side of CCI rats (Scale 500 um, p > 0.05 vs Contralateral). Right column of images: higher power magnification of a section of the image on the left. **B** Double staining showed that SPOCK2 expression in astrocytes (GFAP) was increased significantly on the ipsilateral side of CCI rats (Scale 500 um, **p < 0.01 vs Contralateral). Right column of images: higher power magnification of a section of the image on the left

Considering that glial cell activation plays an important role in the development of NP, we detected the expression and localization of SPOCK2 in microglia and astrocytes after CCI using immunofluorescence. CC11B and GFAP were used as markers of microglia and astrocytes, respectively. At 14 days after CCI, the expression of CD11B and GFAP increased significantly in the dorsal horn, which reflected the increased activation of microglia and astrocytes (Fig. 4A, B). Double immunofluorescence staining showed that SPOCK2 colocalized mainly with astrocytes (Fig. 4B) and less with microglia (Fig. 4A) on the contralateral side of CCI rats. On the ipsilateral side, there was a significant increase in SPOCK2 expression in astrocytes (GFAP) (p < 0.01), while no significant change was observed in microglia. Thus, we speculate that SPOCK2 may participate in NP by affecting astrocytic activation.

**Fig. 5:**
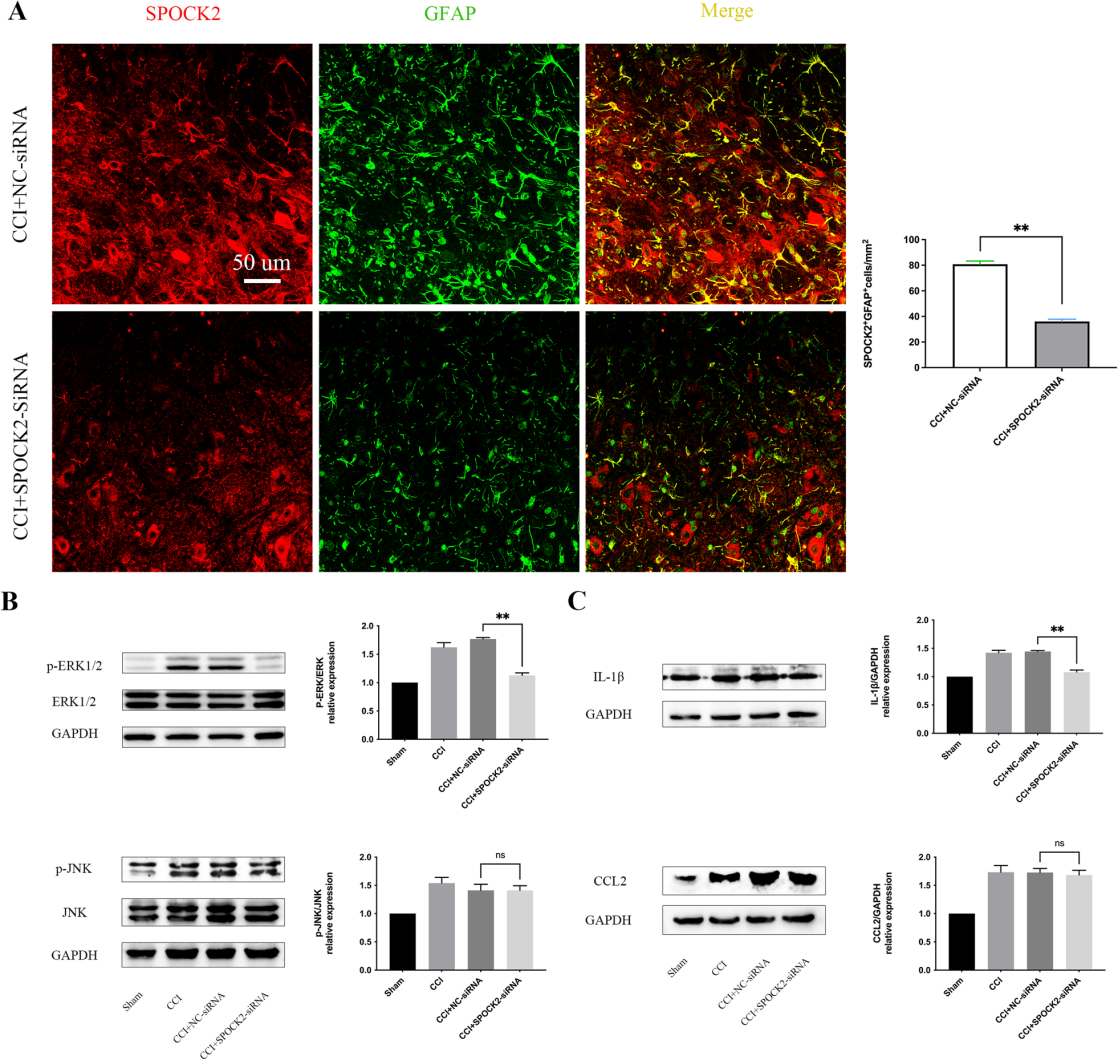
RNAi-mediated SPOCK2 downregulation in CCI rats reduces astrocytic activation, ERK1/2 activation and IL-1β production. **A** Double staining showing decreased (**P < 0.01 vs CCI + NC-siRNA) colocalization of SPOCK2 with GFAP after i.t. injection of SPOCK2-siRNA (Scale 50 um). **B** Decreased expression ratio of p-ERK/ERK after i.t. injection of SPOCK2-siRNA. (**P < 0.01 vs CCI + NC-siRNA). The expression ratio changes of p-JNK/JNK were not significant (P > 0.05 vs CCI + NC-siRNA) after i.t. injection of SPOCK2-siRNA. **C** Decreased expression level of IL-1β after i.t. injection of SPOCK2-siRNA. (**P < 0.01 vs CCI + NC-siRNA). The change in expression of CCL2 was not significant (P > 0.05 vs CCI + NC-siRNA) after i.t. injection of SPOCK2-siRNA

To verify our hypothesis, we sought to identify whether the downregulation of SPOCK2 in the spinal cord would alter the status of astrocyte after CCI as described in *Protocol III*. As shown in Fig. 5A, double immunofluorescence staining showed significantly decreased (p < 0.01) colocalization of SPOCK2 with GFAP after i.t. injection of SPOCK2-siRNA in the rat CCI model. Based on the above results, SPOCK2 could modulate NP by affecting astrocyte activation.

### SPOCK2 modulates NP by affecting ERK1/2 activation and IL-1β production

Considering that alterations in JNK and ERK1/2 pathways play an important role in astrocyte activation when NP occurs [36, 37], we first examined the phosphorylation level of ERK1/2 and JNK after i.t. injection of SPOCK2-siRNA in the rat CCI model as described in *Protocol III*. As shown in Fig. 5B, the increased expression of SPOCK2 in the CCI group coincided with elevated p-ERK/ERK and p-JNK/JNK ratio, whereas the i.t. injection of SPOCK2-siRNA reduced the p-ERK/ERK ratio (p < 0.01). However, the p-JNK/JNK ratio did not show significant changes (p > 0.05). In addition, we detected the expression of pro-inflammatory cytokines (IL-1β) and chemokines (CCL2) in the rat spinal cord after i.t. injection of SPOCK2-siRNA in the CCI model. As shown in Fig. 5C, the expression of IL-1β in the rat spinal cord significantly decreased (p < 0.01) in the CCI + SPOCK2-siRNA group. However, the expression of CCL2 in the rat spinal cord did not show a significant difference (p > 0.05) between the CCI + NC-siRNA and CCI + SPOCK2-siRNA groups. Taken together, SPOCK2 downregulation alleviated NP by reducing ERK1/2 activation and IL-1β production.

### SPOCK2 acts as an upstream molecule to regulate MMP-2 activation involvement in NP

**Fig. 6:**
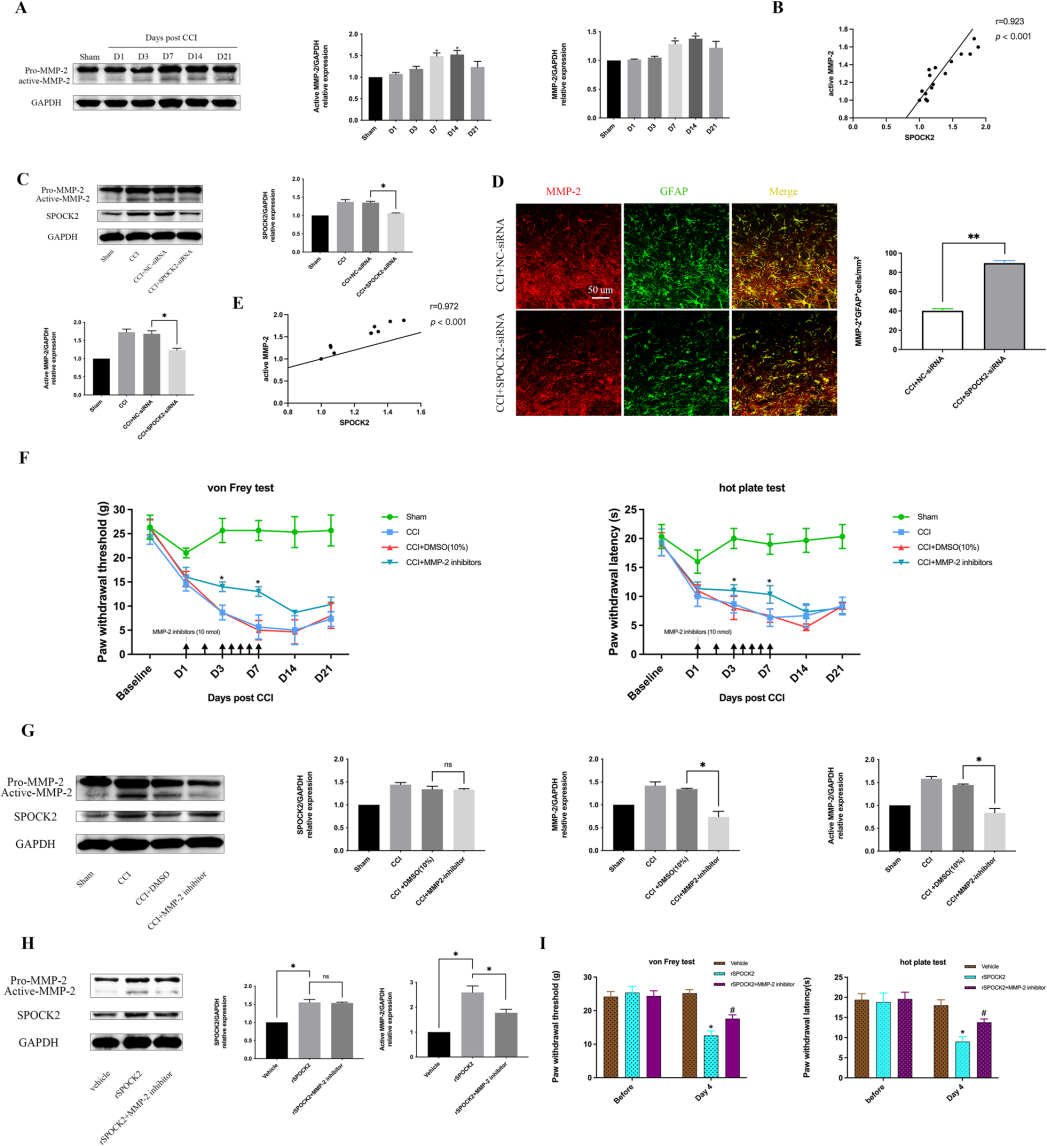
SPOCK2 acts as an upstream molecule to regulate the MMP-2 activation in the development of NP. **A** Time course of changes in MMP-2 expression and activation in the spinal cord in the sham group at 1, 3, 7, 14, and 21 days after CCI operation. The sham operation group was used as control. **B** Pearson correlation analysis to examine the correlation between SPOCK2 and active-MMP-2 in the spinal cord after CCI (r > 0, p < 0.001). **C** Decreased expression level of SPOCK2 and active-MMP-2 after i.t. injection of SPOCK2-siRNA. (*P < 0.05 vs CCI + NC-siRNA). **D** Double staining showing decreased (**P < 0.01 vs CCI + NC-siRNA) colocalization of MMP-2 with GFAP after i.t. injection of SPOCK2-siRNA (Scale 50 μm). **E** Pearson correlation analysis to examine the correlation between SPOCK2 and active-MMP-2 in the spinal cord after i.t. injection of SPOCK2-siRNA in the rat CCI model (r > 0, p < 0.001). **F** Continuous i.t. injection of MMP-2 inhibitors for 7 days (from CCI 1 to 7 days) increased PWT and PWL at 3 and 7 days after CCI (*P < 0.05 vs CCI + DMSO [10%]). **G** Decreased expression and activation of MMP-2 after i.t. injection of MMP-2 inhibitors. (*P < 0.05 vs CCI + DMSO [10%]). The change in expression of SPOCK2 was not significant (P > 0.05 vs CCI + DMSO [10%]) after i.t. injection of MMP-2 inhibitors. **H** Increased expression of SPOCK2 and active-MMP-2 after i.t. injection of rSPOCK2 in SD rats. (*P < 0.05 vs Vehicle). Decreased expression of active-MMP-2 after i.t injection of rSPOCK2 followed by i.t. injection of MMP-2 inhibitors (*P < 0.05 vs rSPOCK2), but the change in expression of SPOCK2 was not significant (P > 0.05 vs rSPOCK2). **I** i.t. injection of rSPOCK2 (0.3 ng, twice daily) in SD rats decreased PWT and PWL at 16 h (Day 4) after the end of injection (*P < 0.05 vs Vehicle). In addition, PWT and PWL showed relatively increased in the rSPOCK2 + MMP-2 inhibitor group (#P < 0.05 vs rSPOCK2)

MMP-2 plays an important role in the maintenance of NP by regulating astrocytic ERK1/2 activation and IL-1β production [30]. Considering that SPOCK2 can be involved in the regulation of MMPs, we hypothesized that SPOCK2 and MMP-2 might be related in the occurrence of NP. Firstly, we detected MMP-2 expression and activation in the rat spinal cord after CCI-induced NP. As shown in Fig. 6A, expression and activation of MMP-2 were increased in the rat spinal cord at 7 and 14 days after CCI-induced NP (p < 0.05). In addition, Pearson correlation analysis showed that the expression of SPOCK2 and active-MMP-2 in the spinal cord were positively correlated (r = 0.923, p < 0.001) after CCI-induced NP (Fig. 6B).

Furthermore, we examined the expression and activation of MMP-2 after i.t. injection of SPOCK2-siRNA in the rat CCI model as described in *Protocol III*. As shown in Fig. 6C, the active-MMP-2 in the rat spinal cord was synchronously downregulated in the CCI + SPOCK2-siRNA group (p < 0.05). Double immunofluorescence staining showed significantly decreased (p < 0.01) colocalization of MMP-2 with GFAP (Fig. 6D), which suggests that SPOCK2 downregulation reduced the MMP-2 activation in astrocytes. Pearson correlation analysis showed that the expression of SPOCK2 and active-MMP-2 in the spinal cord were also positively correlated (r = 0.972, p < 0.001) after i.t. injection of SPOCK2-siRNA in the rat CCI model (Fig. 6E).

In addition, we intrathecally injected 10 nmol MMP-2 inhibitors for seven continuous days (after CCI, 1–7 days) to downregulate MMP-2 expression and activation in the spinal cord as described in *Protocol IV*. The results showed that mechanical allodynia (PWT) and thermal hyperalgesia (PWL) significantly increased following decreasing MMP-2 activation (p < 0.05) in the CCI + MMP-2 inhibitor group (Fig. 6F). However, the SPOCK2 expression level did not significantly differ (p > 0.05) between the CCI + DMSO (10%) and CCI + MMP-2 inhibitor groups (Fig. 6G).

To further explore the relationship between SPOCK2 and MMP-2 in the development of NP, we i.t. injected rSPOCK2 or MMP-2 inhibitor in SD rats as described in *Protocol V*. The results showed that repeated i.t. injection of rSPOCK2 upregulated SPOCK2 expression (p < 0.05) and MMP-2 activation (p < 0.05) and decreased the PWT (p < 0.05) and PWL (p < 0.05) in the rSPOCK2 group compared with the Vehicle group, which suggests that repeated i.t. injection of rSPOCK2 in the spinal cord could induce NP. However, i.t. injection of MMP-2 inhibitor could downregulate rSPOCK2-induced MMP-2 activation (p < 0.05), but the SPOCK2 expression level did not significantly differ (p > 0.05). Additionally, compared with the rSPOCK2 group, the PWT (p < 0.05) and PWL (p < 0.05) showed relative increase in the rSPOCK2 + MMP-2 inhibitor group, which suggests that MMP-2 inhibitor could suppress rSPOCK2-induced NP.

Therefore, we could assume that SPOCK2 can act as an upstreasm molecule to influence NP by regulating astrocytic MMP-2 activation.

### SPOCK2 interacts with MT1-MMP to regulate the activation of MMP-2 by SPARC_EC domain

**Fig. 7:**
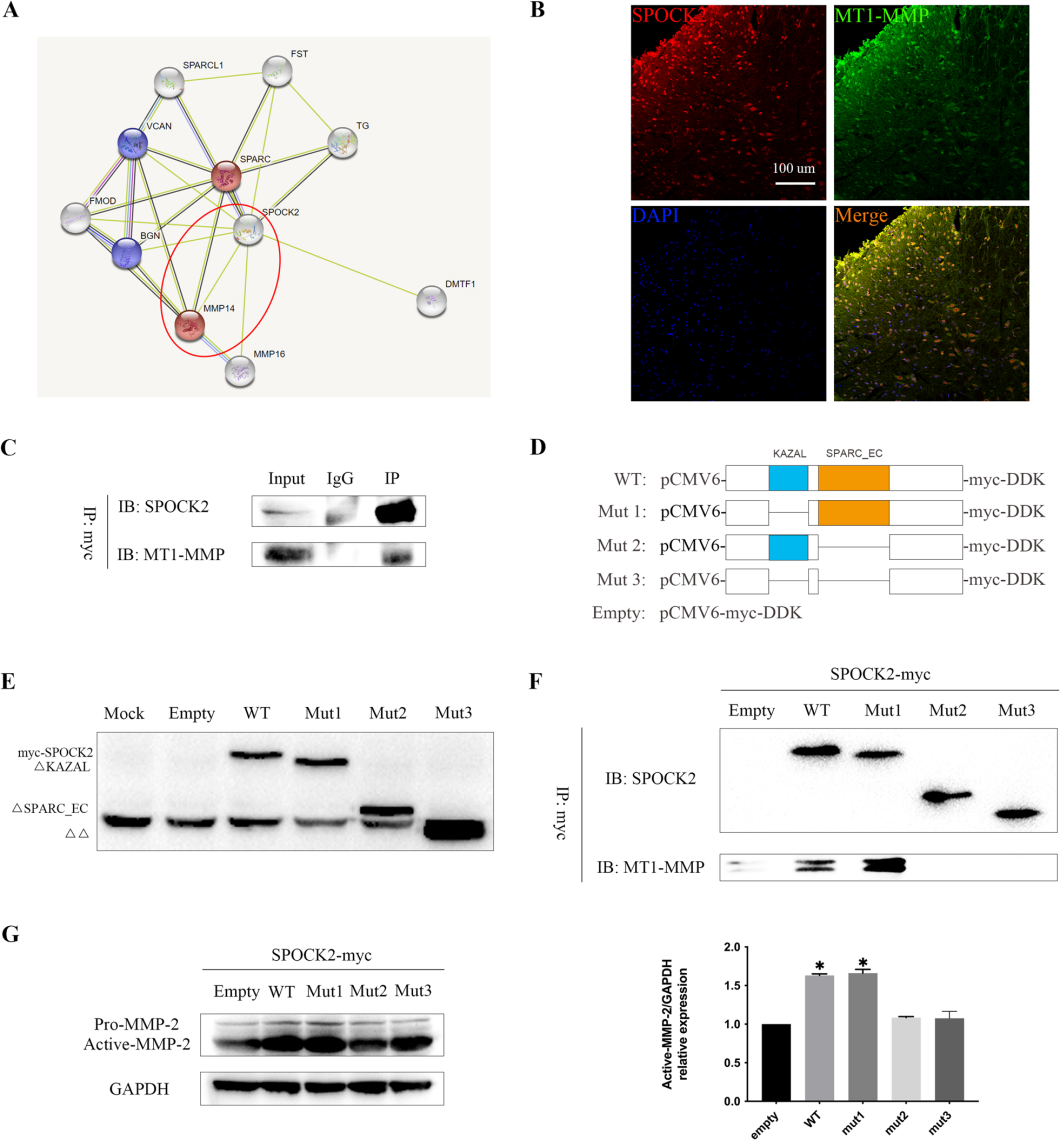
SPOCK2 interacts with MT1-MMP to regulate the activation of MMP-2 by the SPARC_EC domain. **A** PPI prediction website STRING showed potential PPI between SPOCK2 and MT1-MMP. **B** Double immunofluorescent staining showed colocalization of SPOCK2 and MT1-MMP in the rat spinal dorsal horn after CCI-induced NP (Scale 100 um). **C** Co-IP of SPOCK2 and endogenous MT1-MMP in HEK-293 T cells transfected with pCMV6-SPOCK2-myc-DDK. **D** Schematic diagram of five SPOCK2 variant plasmids. **E** Transfection efficiency of plasmids pCMV6-myc-DDK, pCMV6-SPOCK2-myc-DDK, pCMV6-ΔKAZAL-myc-DDK, pCMV6-ΔSPARC_EC-myc-DDK, and pCMV6-ΔΔ-myc-DDK in HEK-293 T cells. **F** Co-IP of SPOCK2 and endogenous MT1-MMP in HEK-293 T cells transfected with five SPOCK2 variants. **G** Increased expression of active-MMP-2 in HEK-293 T cells transfected with pCMV6-SPOCK2-myc-DDK and pCMV6-ΔKAZAL-myc-DDK plasmids (*P < 0.05 vs empty)

To further explore how SPOCK2 affects MMP-2 activation, we founded the intermediate bridge, MT1-MMP. Firstly, MT1-MMP is a key enzyme for pro-MMP-2 activation [28]. Furthermore, considering that SPOCK2 and MT1-MMP are both multi-domain proteins, we suspect that there maybe an interanction between them. Among several protential protein–protein interaction (PPI) networks of SPOCK2 in STRING database (https://string-db.org/), we did find MMP-14 (MT1-MMP) (Fig. 7A). Considering that We then investigated the expression and colocalization of SPOCK2 and MT1-MMP in rat spinal dorsal horn after CCI-induced NP. Double immunofluorescent staining showed that SPOCK2 and MT1-MMP were widely expressed in rat spinal dorsal horn and were co-locolized (Fig. 7B). These results suggested that SPOCK2 may interact with MT1-MMP to regulate activation of MMP-2. To prove our hypothesis, HEK-293 T cells were used as a tool cell for in vitro experimental studies*.* Co-IP experiments revealed that SPOCK2 interacts with MTI-MMP in HEK-293 T cells after transfecting with WT-SPOCK2 plasmid (Fig. 7C). Together with fluorescent colocalization of these two proteins in the rat dorsal horn, these data support the PPI between SPOCK2 and MT1-MMP. To further explore the exact function domain, five SPOCK2 mutants were constructed to investigate the interaction domains (Fig. 7D). Based on the premise that the transfection efficiency in HEK-293 T cells is ensured (Fig. 7E), Co-IP experiments in HEK-293t cells transfected with WT-SPOCK2 and ΔKAZAL plasmids showed an interaction between SPOCK2 and MT1-MMP, and that the interaction was no longer occurred in cells transfected with control plasmid or ΔSPARC_EC or ΔΔ plasmids (Fig. 7F). In addition, western blot analysis demonstrated that WT-SPOCK2 and ΔKAZAL plasmid transfection could upregulate the active-MMP-2 expression levels (p < 0.05) (Fig. 7G). Based on the above results, we conclude that SPOCK2 interacts with MTI-MMP to affect the activation of MMP-2 by the SPARC_EC domain.

## Discussion

To the best of our knowledge, this is the first study on the role and mechanism of SPOCK2 in the pathogenesis of NP. SPOCK2 protein levels were markedly increased in the spinal cord after CCI-induced NP, especially on the 7th and 14th days after sugery, SPOCK2 expression peaked along with CCI-induced pain behaviors (PWT and PWL). In order to better understand the role of SPOCK2 in NP, we constructed two experimental modes to reduce SPOCK2 expression. Firstly, we transfected lentiviral vectors carrying SPOCK2-shRNA to downregulate SPOCK2 expression before CCI sugery, rats behavior tests showed the significant changes of mechanical allodynia (PWT) and thermal hyperalgesia (PWL) in the CCI + SPOCK2-shRNA group. This result demonstrates SPOCK2 can participate in the process of NP. In addition, when siRNA mediated the knock-down of SPOCK2 in the spinal cord after CCI, the pain behaviorl tests (PWT and PWL) were alwo significantly changed. In addition, we also upregulated SPOCK2 expression in SD rats by i.t. injection of rSPOCK2. The results showed the significant decrease of PWT and PWL in the rSPOCK2 group, which suggests that i.t. injection of rSPOCK2 protein could induce NP. Overall, these findings indicate that SPOCK2 participates in the progression of NP.

However, the mechanism by which SPOCK2 is involved in NP remains to be explored. Full evidence have demonstrated that spinal microglia and astrocytes play a vital role in the pathogenesis of NP [13–15, 38]. To further explore whether SPOCK2 could affect the activation of glial cells in the development of NP, firstly, we detected the co-localization of SPOCK2 with GFAP and CD11B by immmunofluorence. The results showed that SPOCK2 was expressed in both astrocytes and microglia in the spinal cord dorsal horn, albeit at low levels. Following induction of NP by CCI, there was a significant increase in SPOCK2 expression specifically in astrocytes but not as pronounced in microglia, indicating its involvement in the mechanism of NP related to astrocyte activation, which is consistent with a study on glioma [32]. Furthermore, i.t. injection of SPOCK2-siRNA after CCI-induced NP could downregulate astrocytic activation and decrease SPOCK2 expression in astrocytes. Based on the above results, we speculated that SPOCK2 may influence astrocytic activation in the development of NP. Considering that astrocytic activation occurs mainly in the late stage of NP, this is precisely corresponding to the correlation between SPOCK2 and chronic BP in the GWAS analysis mentioned above [23]. However, immunofluorescence results revealed some co-localization of SPOCK2 with microglia and neuronal-like cells. And we founded a declining trend in SPOCK2 expression on day 21 after CCI. These suggested that SPOCK2 may also indirectly participate in microglia activation or neuron-related mechanisms in the early stage of NP. Nevertheless, considering that the pathogenesis of NP is relatively complex, the mechanisms involved in microglia activation and neuron are not explored in our study. Investigating whether SPOCK2 can indirectly influence microglial activation or neuron in the early stage of NP will be our focus for future research.

Mitogen-activated protein kinases (MAPKs) play an important role in the development of NP [39]. The major MAPK family members are ERK1/2, p38 MAPK, JNK, and ERK5[40]. Different MAPKs have varying activation patterns in the development of NP [41–46]. Whereas ERK5 and p38 MAPK are activated in microglia [3, 5, 36, 47, 48], JNK is mainly activated in astrocytes [37] and ERK1/2 is activated early in microglia and later in astrocytes [36]. Our current evidence suggests that SPOCK2 involved in the activation of astrocytes during NP, we mainly examined the activation of JNK and ERK1/2 after RNAi-mediated downregulation of SPOCK2 in the spinal cord after CCI. The results showed that downregulation of SPOCK2 could reduce ERK1/2 activation in astrocytes, but not alter the activation of JNK.

NP caused by nerve injury can induce the activation of immune cells and glial cells to release various inflammatory mediators in the spinal cord, such as cytokines and chemokines [49–51]. Our study found that RNAi-mediated SPOCK2 downregulation in rat spinal cords after CCI reduced IL-1β production in the spinal cord, but did not alter CCL2 production. In addition, previous studies [52, 53] have proven that JNK-mediated astrocytic activation could increase CCL2 expression. In the present study, the above results indicate that SPOCK2 affected astrocytic activation in the development of NP through the ERK1/2 signaling pathway rather than JNK/CCL2 signaling pathway.

Recent studies [30, 31, 54] have shown that MMP-2 plays an important role in the development of NP by regulating astrocytic ERK1/2 activation and IL-1β production. In addition, as mentioned above, evidence indicates that SPOCK family proteins are involved in the regulation of MMPs. Therefore, we can speculate that SPOCK2 can regulate the activation of MMP-2 to affect astrocytic ERK1/2 activation and IL-1β cleavage in the development of NP. Firstly, we founded that MMP-2 expression and activation levels were markedly increased after CCI-induced NP. Moreover, the expression trend of active-MMP-2 was consistent with that of SPOCK2 at all time points after CCI. Pearson correlation analysis showed that the expression of SPOCK2 and active-MMP-2 in the spinal cord were positively correlated after CCI-induced NP.

To further explore the relationship between SPOCK2 and MMP-2 in the development of NP, firstly, we seperately downregulated SPOCK2 and MMP-2 expression to detect both proteins’ level in the rat spinal cord after CCI surgery. The results showed that RNAi-mediated SPOCK2 downregulation in the spinal cord after CCI could synchronously downregulate the activation of MMP-2. Pearson correlation analysis showed that the expression of SPOCK2 and active-MMP-2 in the spinal cord were also positively correlated after i.t. injection of SPOCK2-siRNA in the rat CCI model. However, ARP100-mediated downregulation of MMP-2 expression and activation in the spinal cord after CCI also relieved pain significantly, but there was no significant change in SPOCK2 expression. Additionally, when repeated i.t. injection of rSPOCK2 promoted the upregulation of SPOCK2 expression and induced NP, MMP-2 activation also increased significantly, which suggests that i.t. injection of rSPOCK2 could induce NP by regulating MMP-2 activation. Moreover, we founded that MMP-2 inhibitor could suppress rSPOCK2-induced NP and decrease MMP-2 activation without affecting SPOCK2 expression. Overall, these results suggest that SPOCK2 may act as an upstream signaling molecule to regulate the activation of MMP-2 in the development of NP.

The question remains as to how SPOCK2 could regulate the activation of MMP-2. Studies have confirmed that MMP-2 activation is mainly regulated by tissue inhibitors of metalloproteinase-2 (TIMP-2) [28, 55], but MT1-MMP is also a key enzyme for pro-MMP-2 activation [28]. In addition, both MT1-MMP and SPOCK2 are multidomain proteins, which provide the structural basis for PPI between them. We also found potential PPI between SPOCK2 and MT1-MMP in STRING. By investigating each domain of SPOCK2, we found two potentially important domains: SPARC_EC domain or KAZAL domain. A previous study [56] pointed out that SPARC could upregulate the expression of MT1-MMP in U87MG glioma cells and then affect the activation of MMP-2, thus promoting the invasion of glioma. Another study [57] showed that Reversion Inducing Cysteine Rich Protein with KAZAL motif (RECK) could increase cell-associated collagenic enzyme activity by promoting MT1-MMP expression. Based on the above research, we hypothesized that SPOCK2 could interact with MT1-MMP to regulate activation of MMP-2 by the SPARC_EC domain or KAZAL domain. The colocalization of SPOCK2 and MT1-MMP in rat spinal dorsal horn after CCI-induced NP provided the basis for our hypothesis. Moreover, we verified our hypothesis in vitro experiments*.* After HEK-293 T cells were transfected with WT SPOCK2 plasmid, Co-IP experiments revealed that SPOCK2 could directly or indirectly combine with endogenous MT1-MMP in HEK-293 T cells. To verify that SPOCK2 can interact with MT1-MMP to regulate activation of MMP-2, Co-IP experiments were performed to separately detect the interaction between five SPOCK2 variants (empty, WT SPOCK2, mut1, mut2, mut3) and endogenous MT1-MMP in HEK-293 T cells. We detected the expression and activation of endogenous MMP-2 in HEK-293 T cells after transfecting with all five SPOCK2 variants. The above results indicate that SPOCK2 could interact with MT1-MMP to regulate activation of MMP-2 by the SPARC_EC domain.

Of note, we assessed the interaction between SPOCK2 and MT1-MMP affecting the activation of MMP-2 only in vitro. Due to the limited conditions, we did not verify our conclusions by performing in vivo experiments. Thus, we will investigate these points through in vivo experiments in the future. Whenever the mechanism underlying SPOCK2 effects can be confirmed in vivo, new ways to relieve NP could be proposed, such as interfering with SPOCK2 and MT1-MMP interaction and their effects on MMP-2 activation.

Additionally, a major limitation of the present study should be acknowledged. The present study was conducted in male animals only. Previous studies have shown sex differences in pain processing and pain modulation at the molecular, cellular, and systems levels and that females have higher pain intensity than males [58, 59]. Further studies in female animals are needed to determine whether sex differences exist regarding the role of SPOCK2 in modulating NP.

## Conclusion

**Fig. 8:**
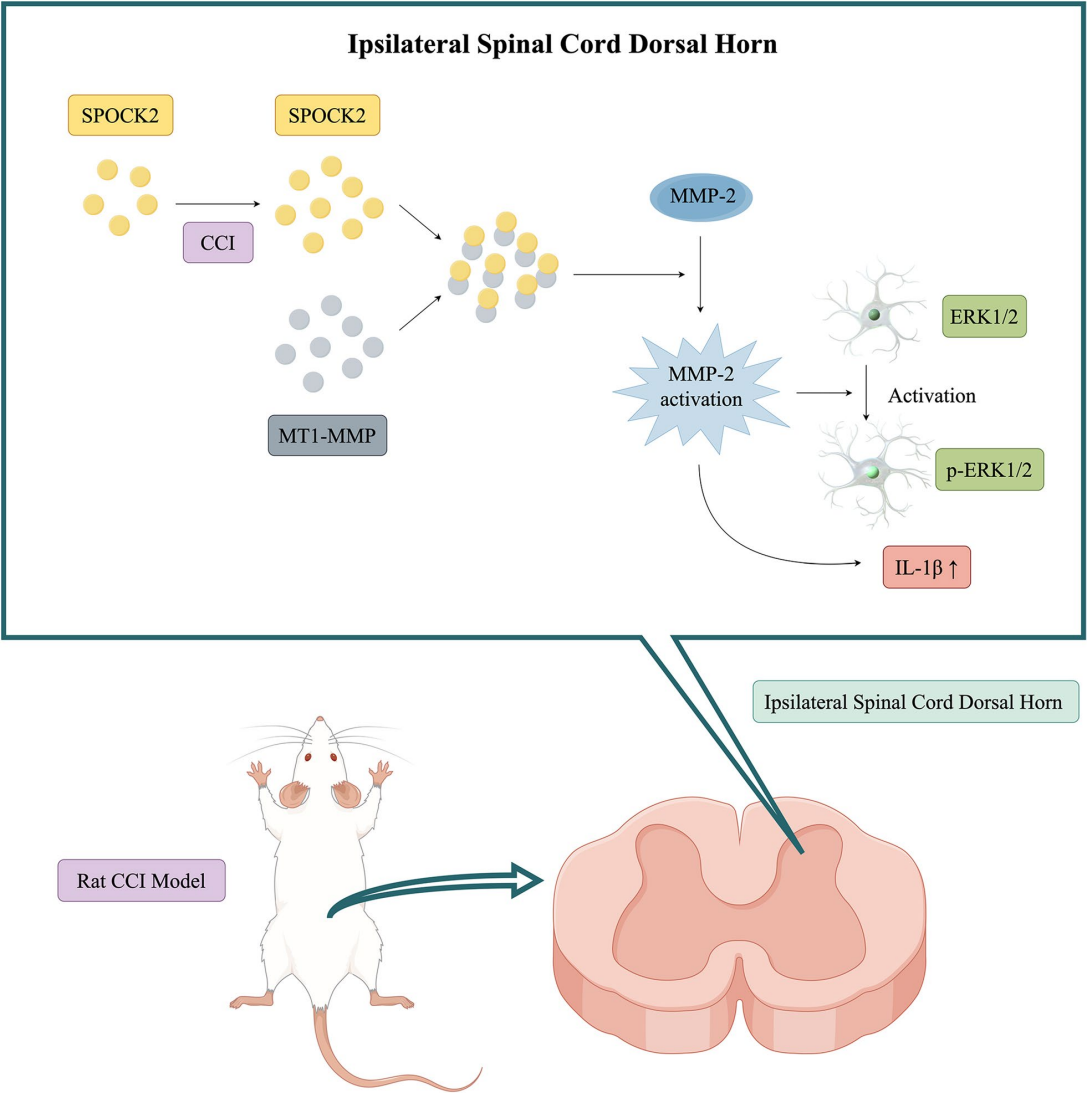
Schematic diagram of SPOCK2 involvement in NP (By Figdraw)

In summary, we demonstrated that SPOCK2 could be a modulator of NP. When nerve injury occurs, SPOCK2 expression is upregulated in the spinal cord. As shown in Fig. 8, SPOCK2 may interact with MT1-MMP to regulate astrocytic MMP-2 activation, thus affecting astrocytic ERK1/2 activation and IL-1β production, ultimately involvment in the development of NP. Our findings provided a new perspective on the pathogenesis of NP.

## Data Availability

The datasets used and/or analyzed in the current study are available from the corresponding author on reasonable request.

## Abbreviations

BP: Back pain
CCL2: CC-chemokine ligand 2
CSF: Cerebral spinal fluid
CCI: Chronic constriction injury
JNK: C-Jun N-terminal kinase
DMSO: Dimethyl sulfoxide
ERK1/2: Extracellular regulated protein kinases 1 and 2
GWAS: Genome-wide association study
GFAP: Glial fibrillary acidic protein
GAPDH: Glyceraldehyde-3-phosphate dehydrogenase
IL-1β: Interleukin-1β
MMP-2: Matrix metalloproteinase-2
MMPs: Matrix metalloproteinases
MT1-MMP/MMP-14: Membrane-type 1 matrix metalloproteinase
MAPKs: Mitogen-activated protein kinases
NC-siRNA: Negative control siRNA
NP: Neuropathic pain
NS: Normal saline
PWL: Paw withdrawal latency
PWT: Paw withdrawal threshold
PVDF: Polyvinylidene difluoride
PPI: Protein–protein interaction
rSPOCK2: Recombinant SPOCK2
RECK: Reversion Inducing Cysteine Rich Protein with KAZAL motif
shScr: Scrambled shRNA
SPOCK2: SPARC/osteonectin, cwcv, and kazal-like domains proteoglycan 2
TIMP-2: Tissue inhibitor of metalloproteinase-2
WT: Wild-type
